# Co-Expression of Neighboring Genes in the Zebrafish (*Danio rerio*) Genome

**DOI:** 10.3390/ijms10083658

**Published:** 2009-08-21

**Authors:** Huai-Kuang Tsai, Pei-Ying Huang, Cheng-Yan Kao, Daryi Wang

**Affiliations:** 1 Institute of Information Science, Academia Sinica, 128 Sec. 2, Academia Rd, Nankang, 115, Taipei, Taiwan; E-Mail: hktsai@iis.sinica.edu.tw (H.-K.T.); 2 Department of Computer Science and Information Engineering, National Taiwan University, Taipei 106, Taiwan; E-Mails: d96004@csie.ntu.edu.tw (P.-Y.H.); cykao@csie.ntu.edu.tw (C.-Y.K.); 3 Biodiversity Research Center, Academia Sinica, 128 Sec. 2, Academia Rd, Nankang, 115, Taipei, Taiwan

**Keywords:** gene expression, co-expression, neighboring genes, promoter, zebrafish

## Abstract

Neighboring genes in the eukaryotic genome have a tendency to express concurrently, and the proximity of two adjacent genes is often considered a possible explanation for their co-expression behavior. However, the actual contribution of the physical distance between two genes to their co-expression behavior has yet to be defined. To further investigate this issue, we studied the co-expression of neighboring genes in zebrafish, which has a compact genome and has experienced a whole genome duplication event. Our analysis shows that the proportion of highly co-expressed neighboring pairs (Pearson’s correlation coefficient R>0.7) is low (0.24% ~ 0.67%); however, it is still significantly higher than that of random pairs. In particular, the statistical result implies that the co-expression tendency of neighboring pairs is negatively correlated with their physical distance. Our findings therefore suggest that physical distance may play an important role in the co-expression of neighboring genes. Possible mechanisms related to the neighboring genes’ co-expression are also discussed.

## 1. Introduction

The distribution of genes in eukaryotic genomes was long believed to be random; however, recent studies have indicated that this is not always the case [1–3]. For example, in the human genome, housekeeping genes show a strong tendency to cluster together [4], and genes that participate on the same pathway also tend to lie adjacent to each other in the genome [3,5,6]. Moreover, several studies indicate that adjacent genes in humans seem to co-express, regardless of their intergenic distance [7–9]. Similar phenomena have been observed in *Drosophila*, nematodes, and yeast [10–14]. Among these observations, the co-expression of adjacent pairs is crucial because changes in the genome’s organization could alter the co-regulated transcription of the pairs [9,12].

The mechanism responsible for the co-expression of adjacent genes is not well understood [3,15]. It has been noted that sharing the regulatory system cannot fully explain the co-expression of adjacent genes [16]. Therefore, investigation of neighboring genes may provide information that will help researchers better understand the gene co-expression mechanism. Recently, based on Pearson’s correlation coefficient (R) values, co-expressions of neighboring genes have been investigated using sliding windows of a certain sequence length (the number of nucleotides) [4] or a given number of genes [13,17,18]. Cohen *et al*. [19] found that yeast genes of neighboring gene triplets display similar expression patterns, while Spellman and Rubin [13] reported that more than 20% of the genes in the *Drosophila* genome are clustered into co-regulated groups of 10–30 genes. In nematodes, many neighboring gene pairs within a distance range of 20 kbp show correlated expressions [17]; and co-expression of neighboring genes has even been observed in plants, such as *Arabidopsis* [20,21]. However, in zebrafish (*Danio rerio*), which is characterized by a compact genome [22], the co-expression tendency of neighboring genes has yet to be investigated. Therefore, in the following, we attempt to fill this research gap.

To investigate whether co-expressed clusters of genes exist in zebrafish, we first examined the co-expression levels among three neighboring gene patterns, namely pairs, triplets and quadruplets. Studying the co-expression of neighboring genes is of particular interest because it is believed that the zebrafish genome may contain about 30% more genes than the human genome, and an additional round of genome duplication occurred about 450 million years ago [23]. Thus, in theory, the duplicated neighboring relationships in zebrafish should be free from evolutionary selection. Recent advances in technology have led to the establishment of databases of whole genome sequences, genomic mapping data with physical distances, and microarray expression profiles. These tools allow researchers to investigate the co-expression mechanism of neighboring genes by studying their proximity and the physical distance between them. In this paper, we investigated whether the co-expressed clusters are associated with the physical distance of the chromosome. Our study provides clear evidence that the physical distance is an essential factor in co-expression of neighboring genes in zebrafish.

## 2. Results and Discussion

### 2.1. Co-Expression of Neighboring Gene Pairs

**Figure 1.**
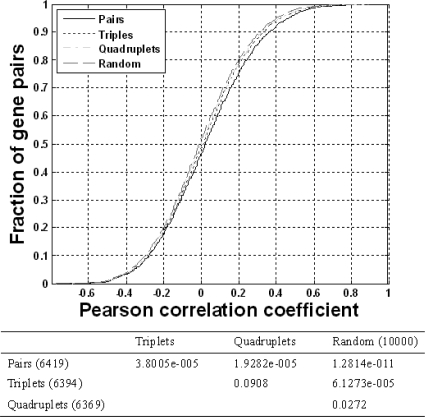
Comparison of the co-expression levels of three neighboring gene patterns (pairs, triplets, and quadruplets). In the upper figure, the Pearson correlation values of the three patterns and those of random pairs are used to construct their individual cumulative distributions. The lower table indicates the significance score of the KS test (*p* value).

To investigate the co-expression of neighboring genes, we combined the NCBI annotation of the zebrafish genome and thirteen microarray datasets from ArrayExpress [24]. Of the 6,444 genes with expression values and their relative positions in the zebrafish genome, we found that only a small fraction of the gene pairs exhibited significant co-expression levels. Specifically, the proportions were 0.67% for adjacent pairs, 0.48% for triplets, and 0.24% for quadruplets, as shown in Table 1 (adjacent pairs, triplets and quadruplets are defined in Materials and Methods). However, as shown in Figure 1, adjacent and triplet pairs exhibited a significant co-expression tendency compared to random pairs (*p*<0.01, one-sided KS test), while quadruplet pairs only exhibited a marginal significance (*p*=0.0272). As expected, the adjacent pairs showed the highest co-expression levels of all the groups.

### 2.2. The Physical Distance between Genes in the Local Co-Expression Domain

Since the co-expression level of neighboring genes in a small domain is significantly higher than that of the random samples, it would be interesting to investigate whether the co-expression level is related to the physical distance between neighboring genes. First, our calculations showed that the mean TLS (translation start site) distances between highly co-expressed neighboring genes (*i.e.,* adjacent pairs, triplets and quadruplets) were remarkably shorter than those of all neighboring genes in the zebrafish genome. The mean distance of highly co-expressed genes differed by 186 kbp (208 kbp versus 394 kbp) compared to that of all neighboring genes, as shown in Table 2. Thus, the results suggest that, since co-expressed neighboring genes are more tightly clustered within the genome, the proximity of highly co-expressed neighboring genes may be an important factor in their co-expression.

**Figure 2.**
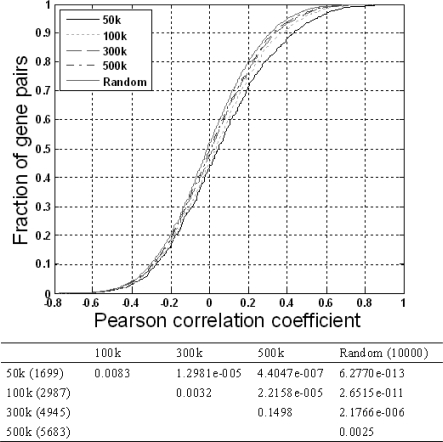
Comparison of the co-expression levels of four gene distance patterns (50 kbp, 100 kbp, 300 kbp and 500 kbp sliding windows). In the upper figure, the Pearson correlation values of the four patterns and those of random pairs are used to construct their individual cumulative distributions. The lower table indicates the significance score of the KS test (*p* value).

Second, we also investigated the co-expression tendency of neighboring genes with various physical distances in the chromosomes. The sizes of the neighboring genes were identified using 50 kbp, 100 kbp, 300 kbp and 500 kbp sliding windows (see Materials and Methods for details). As shown in Figure 2, the co-expressions of four groups, 50 kbp (1699 pairs), 100 kbp (2987 pairs), 300 kbp (4945 pairs) and 500 kbp (5683 pairs) exhibit significantly higher co-expression levels compared to those of random pairs (*p*<0.01, one-sided KS test). The statistical results in Figure 2 also indicate a negative correlation between co-expression and distance, which implies that the physical distance between co-expressed pairs in a chromosome may have an effect on the co-expression level.

### 2.3. Characterization of the Local Co-Expression Domain

**Figure 3.**
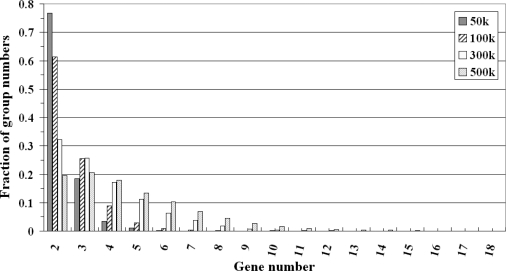
Distribution of group sizes for various physical distances (50 kbp, 100 kbp, 300 kbp and 500 kbp sliding windows).

To characterize the sliding windows (50 kbp, 100 kbp, 300 kbp and 500 kbp), the number of genes in each group (sliding window) were counted for comparison. Figure 3 shows that, in each sliding window, the dominant components were windows that contained two genes. For example, 76.81% of the groups in the 50 kbp window contained two genes. Similar patterns were observed in the groups in the 100 kbp (61.30%) and 300 kbp (32.30%) windows. However, due to the limitations of gene annotations and genome mapping, the number of genes in a sliding window might be underestimated. Nevertheless, it is likely that adjacent pairs make the largest contribution to co-expression.

### 2.4. Characterization of Adjacent Gene Pairs

**Figure 4.**
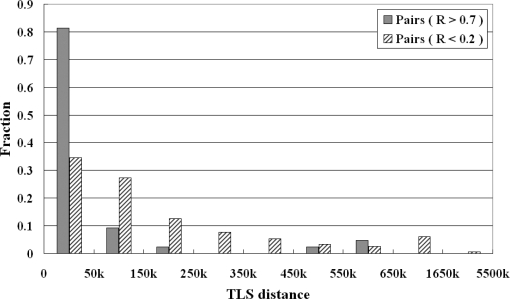
Distribution of TLS distances (kbp) in highly co-expressed adjacent gene pairs (Pearson correlation value >0.7) and lowly co-expressed adjacent gene pairs (Pearson correlation value <0.2).

Since most adjacent pairs are co-expressed, we hypothesize that the genetic distance between adjacent pairs might be a contributory factor to their higher co-expression levels. To characterize the structural features of adjacent gene pairs, we compared the distributions of the TLS distances between adjacent pairs with highly correlated expressions (R>0.7) and those with lowly correlated expressions (R<0.2). Figure 4 shows that the TLS distance of the majority (81.40%) of highly co-expressed adjacent gene pairs is less than 50 kbp, and the distribution drops sharply as the distance increases. A statistical test (the Chi square-test, *p*<0.001) showed that the distributions of the highly co-expressed group were significantly different from those of the lowly co-expressed group. Hence, it might be inferred that gene pairs with a shorter distance have a higher co-expression tendency.

### 2.5. The Role of Tandem Repeat Pairs in Neighboring Gene Pairs

**Figure 5.**
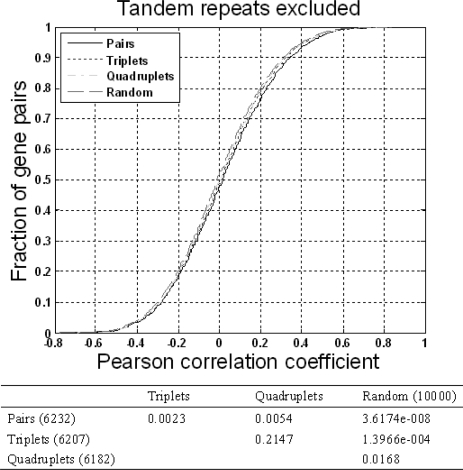
Comparison of the co-expression levels of the three neighboring gene patterns without tandem repeats. The lower table indicates the significance score of the KS test (*p* value).

To eliminate bias due to tandem repeat genes, we excluded such genes and repeated all the above tests. Adjacent pairs (6,232) and triplet pairs (6,207) still exhibited a significant co-expression level compared to that of random pairs (*p*<0.01, one-sided KS test), while quadruplet pairs (6,182) showed a marginal significance (*p*=0.0168) (Figure 5). The trends were similar in data that included tandem repeats (Figure 1).

### 2.6. Discussion

It is known that some neighboring genes in eukaryotic genomes express concurrently [4,15,18,21,25]. The co-expressed domains vary in their spanning distance, the number of genes and their co-regulation behavior. We hypothesize that if gene adjacency is not restricted by selection, neighboring genes in organisms that had experienced whole-genome duplication (WGD), such as zebrafish, should have lost their neighboring relationships as well as their tendency to co-express. In zebrafish, neighboring genes (adjacent and triplet pairs) have a higher expression correlation than that of random pairs, which suggests the co-expression tendency in neighboring genes, and consequently, the co-expressed gene pairs have retained their neighboring relationships through evolution. A similar phenomenon has been observed in other vertebrates [1,4,25], and also in plants, such as *Arabidopsis* [20]. Moreover, in zebrafish, we found that the proportion of highly co-expressed adjacent pairs (R value >0.7) is higher than the proportion of co-expressed gene pairs in triplets and quadruplets. Based on the hypothesis that the close proximity of neighboring genes could lead to the sharing of *cis*-regulatory elements [18,26], it is likely that such sharing could partially explain gene co-expression behavior in zebrafish.

Two key mechanisms explain the co-expression of neighboring genes: alteration of the chromatin structure and sharing of regulatory elements [1,3,11]. Our analysis demonstrates that genes in close proximity in a chromosome have a stronger tendency to co-express than genes that are farther apart. This finding further supports the hypothesis that the shorter the physical distance between two genes, the higher the probability that the genes will share *cis*-regulatory elements and result in their co-expression [18,20]. This hypothesis is also supported by the work of Fukuoka *et al.* [11], who found that the co-expression levels of genes correlate strongly with the physical distance between the genes rather than with the adjacency of the genes. However, Spellman and Rubin [13] suggested otherwise. In their study, the pattern of gene expression within a neighborhood is independent of the physical distance between gene pairs. Moreover, studies of human genes have shown that co-expression between adjacent genes is not related to their physical distance [7–9]. Nevertheless, more direct evidence is required to determine the mechanisms that govern the co-expression of neighboring genes [15,27].

In the zebrafish genome, we found that, on average, highly co-expressed neighboring genes (R>0.7) were much closer to each other than the neighboring genes that did not exhibit co-expression. This finding suggests that the physical distance between gene pairs is an important factor in their co-expression behavior. The phenomenon of co-expressed clusters has also been observed in other organisms. For example, in the yeast genome, Lercher and Hurst [28] found clusters of local co-expression ranging up to 30 genes (100 kbp); and in the *Arabidopsis* genome, there are local clusters of up to 20 genes that co-express, with an overall median cluster size of 100 kbp [18]. Higher co-expression clusters ranging up to 200 kbp have also been found in the *Drosophia* genome [13]. Surprisingly, the highly co-expressed neighboring genes in zebrafish span a larger distance than other model organisms studied [13,19,21,27]. Although the reason for this difference is still not clear, our observations suggest that chromatin modifications might contribute to co-expression behavior because multigenic loci within an acetylated chromatin can be commonly regulated [29,30].

By using sliding windows up to 500 kbp in size, the significance of co-expressed clusters can be observed in the zebrafish genome. Similarly, in the *Drosophila* genome, 10 to 30 co-expressed genes can be found in clusters by using a 20~200 kbp sliding window [13]. The same pattern is also observed in *C. elegans.* After removing duplicate genes, the significant clusters of co-expressed genes are restricted to distances within 20 kbp [17]. It has been suggested that co-expressed gene clusters that span long distances are consistent with the boundaries of the chromatin structure [31,32]. A recent study also observed that radial chromatin positioning is preferentially shaped by the local gene density [29]. Since the genes within a euchromatic domain might increase the accessibility of the promoters and enhancers of other genes to the transcriptional machinery, thereby leading to co-expression [13,33], it is possible that the larger co-expressed clusters may be partly related to the compact genome of zebrafish [22].

The duplication of promoter elements is a minor factor that might contribute to neighboring gene co-expression [21]. To eliminate the effect of duplicated sequences, we removed tandem repeats from our data. However, since our results show a similar trend after excluding tandem repeats, we suspect that such repeats might not be a major cause of co-expression in the neighboring genes of zebrafish. In addition, physically overlapping genes were excluded from the analysis due to possible bias in measuring the expression levels [12]. Since our analysis excludes tandem repeats, overlapping genes and ambiguous data, we can provide reliable information with limited bias. (See Materials and Methods for details). Under our stringent criteria, we used 6,444 genes for analysis; thus, our results might still underestimate the co-expression of neighboring genes in zebrafish.

## 3. Materials and Methods

### 3.1. Data Preparation

Annotations of 25,168 known and putative Zebrafish (*Danio rerio*) genes were downloaded from the National Center for Biotechnology and Information (NCBI), and their genomic mapping data with physical distances was downloaded from the NCBI Map Viewer (ftp://ftp.ncbi.nih.gov/genomes/MapView/Danio_rerio/sequence/BUILD.3.1/). To eliminate ambiguity in our analysis, we removed 142 dubious genes and 1,868 overlapping genes and ignored the gene order. As a result, we had a total of 23,518 genes for analysis.

Thirteen zebrafish microarray datasets, covering 47 conditions and 14,443 genes, were analyzed. Six zebrafish gene expression datasets, namely, indexed E-TABM-33, E-MEXP-171, E-MEXP-405, E-MEXP-736, E-MEXP-737 and E-MEXP-758, were downloaded from the ArrayExpress database (http://www.ebi.ac.uk/microarray-as/ae/). Seven zebrafish gene expression datasets, namely, indexed GSE1894, GSE1995, GSE3303, GSE3667, GSE4201, GSE4989, and GSE4585, were downloaded from the GEO database (http://www.ncbi.nlm.nih.gov/geo/). The repeated plotted genes for normalization of the microarray were only calculated once. Of the 14,443 genes, 11,352 were mapped to 9,385 distinct gene IDs according to the zebrafish annotations provided by the NCBI database (published in August 2008). After mapping the gene expression data by their unique IDs and the annotations, we obtained 6,444 genes with their expression values and the gene order. Note that putative genes and genes with duplicated annotations or without expression data were not considered. It is also noteworthy that neighboring genes that lie in close proximity to each other on the chromosome are not necessarily adjacent to each other.

We selected thirteen zebrafish microarray datasets for the expression analysis. To reduce the systematic biases within each dataset as well as the intensity-dependent effects and biases between datasets, we used the MA lowess and quantile normalization methods. In this study, we used Pearson’s correlation coefficient (R) to determine the correlation between two gene expression patterns. The value of R is defined as the covariance of the two expression patterns divided by the product of their standard deviations, which reflects the degree of the linear relationship between the two expression patterns. The obtained values range from +1 to −1. A value of +1 (resp. −1) indicates a perfect positive (resp. perfect negative) linear relationship between the two expression patterns, while zero means there is no linear relationship between the expression patterns. Note that the correlation coefficient is unreliable for datasets with small sample sizes. We empirically determined that a dataset should have many time points for a reliable estimation of the correlation coefficients. Therefore, we merged the thirteen datasets into one large dataset for the analysis.

### 3.2. Identification of Co-Expression in Neighboring Genes

To investigate whether neighboring gene pairs have a stronger tendency to co-express, we compared the expression correlations of adjacent pairs, triplets, and quadruplets (sliding windows of 2, 3 and 4 genes respectively) [13] to the expression correlations of a group of 10,000 non-adjacent random pairs. The gene order was determined using the 6,444 genes derived from the genomic mapping and expression datasets. In addition to adjacent pairs, the Pearson coefficient values for triplets and quadruplets were determined by calculating the expression profiles of the first gene and the last gene of a group. This contrasts with the method used by Ren *et al*. [20] who took the average of all pairwise Pearson coefficients, and therefore might have overrepresented the co-expression level. We used the Pearson correlation values of the three neighboring gene patterns (*i.e.,* pairs, triplets, and quadruplets) and random pairs to construct their individual cumulative distributions. Then, based on the respective distributions, we used the one-sided Kolmogorov-Smirnov (KS) test to determine whether two groups of gene pairs co-expressed to different extents. For example, to test whether the distribution of pairs and the distribution of triplets differed significantly, we compared *H*_0_ *: F_pairs_ = F_triplets_* with *H*_1_ *: F_pairs_ < F_triplets_* using the one-sided KS test, where *F* denotes the cumulative distribution function of the Pearson coefficients of a gene expression pattern. If *H_0_* was rejected, *F_pairs_ < F_triplets_*, which meant the Pearson coefficients in a group of pairs were ‘stochastically greater’ than those in a group of triplets.

### 3.3. Identification of Local Co-Expression Levels

To investigate the correlation between the co-expression level and the physical distance of neighboring genes, we compared the expression correlations of genes within certain distances (50 kbp, 100 kbp, 300 kbp, and 500 kbp sliding windows) [4] to the expression correlations of a group of 10,000 non-adjacent random pairs. We then used the one-sided Kolmogorov-Smirnov (KS) test to examine whether two groups of gene pairs co-expressed to different extents based on their cumulative distribution of Pearson coefficients. We define the gene distance as the distance between the TLS of the first gene and the TLS of the last gene in the group.

### 3.4. Determination of Tandem Repeat Genes

To eliminate the possibility of co-expression bias caused by tandem repeat genes, we separated such genes for analysis. We considered a gene pair as a tandemly repeated gene if the local pairwise protein BLASTP yielded E < 2 × 10^−1^ [4,11,17,18]. This criterion is based on the identification of human duplicated genes. The pairwise search which is used to identify human duplicated genes successfully removed about 90% of the related genes in the human population [4,18].

## 4. Conclusions

Our results show that significantly co-expressed clusters of neighboring genes are co-regulated across large distances up to 500 kbp. The co-expressed domains consist of two to four genes. Theoretically, the modification of the chromatin structure can be used to explain the co-expression of neighboring genes in zebrafish, as separated loci within an acetylated chromatin can be commonly regulated. However, we also found that highly co-expressed genes are strongly favored in adjacent pairs, suggesting that the adjacency of neighboring genes may be a major factor in co-expression. Close examination of adjacent pairs shows that co-expressed adjacent pairs span a shorter distance than non- co-expressed adjacent pairs. These observations suggest that the physical distance between adjacent genes plays an important role in their co-expression and consequently links with the second mechanism–sharing of the regulatory elements. Therefore, we conclude that both of the key mechanisms contribute to the co-expression of neighboring genes in the zebrafish genome. In addition, the compact genome of zebrafish might also have an effect on the phenomenon of co-expression; hence, it too merits further investigation.

## Figures and Tables

**Table 1.** The number of co-expression gene pairs in adjacent pair, triplet and quadruplet groups.

	**Total^a^**	**R>0.7 (ratio)^b^**	**R>0.6 (ratio)^c^**	**R>0.5 (ratio)^d^**
Adjacent pairs	6419	43 (0.67%)	98 (1.53%)	250 (3.89%)
Triplets	6394	31 (0.48%)	82 (1.28%)	194 (3.03%)
Quadruplets	6369	15 (0.24%)	70 (1.10%)	184 (2.89%)
Random	10000	18 (0.18%)	76 (0.76%)	220 (2.20%)

**Table 2.** Median and mean physical distances^a^ and standard deviations (std) of neighboring genes in the zebrafish genome.

	**All neighboring genes^b^**	**Highly correlated neighboring genes^c^**	**Lowly correlated neighboring genes^d^**
Median (kbp)	236	33	245
Mean (kbp)	394	208	401
std	473	393	476
Total groups	19182	89	14855

^a^ The physical distances were calculated from the TLS of the first gene to the TLS of the last gene in the group.

^b^ Neighboring gene pairs include adjacent pairs, triplets, and quadruplets.

^c^ The neighboring gene pairs with highly correlated expressions of R>0.7.

^d^ The neighboring gene pairs with lowly correlated expressions of R<0.2.
